# An Ayurvedic Herbal Extract Inhibits *Streptococcus mutans* Biofilm Formation and Disrupts Preformed Biofilms *in vitro*

**Published:** 2020-08-12

**Authors:** Sunethra Rajapakse, Michael A Giardino, Hemantha D Kulasekara, Richard P Darveau, Ana M Chang

**Affiliations:** 1 Department of Oral Medicine and Periodontology, University of Peradeniya, Peradeniya, Sri Lanka; 2 University of the West Indies, St. Augustine, Trinidad and Tobago; 3 Department of Periodontics, University of Washington School of Dentistry, Seattle, WA, USA; 4 Department of Genome Sciences, University of Washington, Seattle, WA, USA

**Keywords:** Herbal, *Streptococcus mutans*, Gingivitis, Oral health, Biofilms, Ayurvedic medicine

## Abstract

**Objective:**

Sudantha® (SUD), a natural proprietary mixture of herbal extracts that has been incorporated into toothpaste, has been shown in two separate placebo controlled human clinical studies to promote gingival health; and reduce gingival bleeding and plaque formation. However, the herbal based anti-gingivitis mechanisms of Sudantha are not fully understood. The objective of this study was to determine the effect of Sudantha on dental plaque biofilms by investigating its effect on mono-culture biofilms of a primary colonizer, *Streptococcus mutans*, *in vitro*.

****Results**:**

This study found that SUD contributes to the maintenance of oral health through the inhibition of *S. mutans* biofilm formation. In addition, SUD disrupted preformed *S. mutans* biofilms after exposure to SUD for 4 hours. Together, this pilot data suggests the inhibition of *S. mutans* biofilm formation and disruption represents one potential mechanism by which the herbal extract is able to reduce the oral bacterial biofilm resulting in its effective against gingivitis and its potential use in countering biofilm associated oral disease.

## Introduction

*Streptococcus mutans* is a normal constituent of the human oral microbiota and is considered to be a primary etiological agent in caries and a primary colonizer of dental biofilms [1,2]. It is an aciduric, facultative anaerobic gram positive cocci equipped with an array of virulence traits such as the ability to adhere to tooth surfaces and form biofilms. Acidogenecity and acidurity, caused by the lactic acid byproducts of sucrose metabolism by *S. mutans*, are significant factors in the etiology and pathogenesis of dental caries [1]. As a commensal member of the oral cavity in health, complete elimination of this organism would not be strategic from an ecological balance point of view. There is increasing recognition that the preservation and/or restoration of the health-associated microbiome are critical to maintain health rather than complete elimination of specific members of the ecosystem in diseased sites or individuals [3,4]. Therefore caries prevention strategies has shifted focus towards targeting virulence factors rather than in the elimination of pathogenic bacteria due to inherent problems associated with antimicrobials such as non-discriminating action affecting all bacteria, development of resistance and superinfections. In a broader sense, it is possible that the use of anti-virulence strategies may exert less stress on the bacteria in the ecosystem so that the drive to develop resistant strains would be reduced. Hence, investigators have directed their efforts towards identification of anti-virulence factors to reduce carcinogenicity of this bacterium.

A variety of natural compounds isolated from plants have been shown to possess activity against major bacterial virulence factors such as quorum sensing, biofilms, motility, toxins and enzymes [5,6]. Sudantha® (SUD) is toothpaste containing a proprietary Ayurvedic blend of plant extracts, which has been used traditionally for oral care [7–13]. In a recently published study it was found that SUD differentially modulated the gingival epithelial cell interleukin-8 (IL-8) response to bacteria and host cytokines [14]. It was postulated that this host associated inflammatory modulation activity of the SUD extract may be contributing to its clinical efficacy in maintaining gingival health as observed in two previously published clinical trials [15,16]. The objective of this study was to determine if SUD also had an effect on dental plaque accumulation separate from the host effects previously published [14]. *Streptococcus mutans* is the principal bacterium associated with dental caries and its accumulation in the supra-gingival plaque biofilm is strongly associated with the development of dental caries (1). Moreover, streptococci are considered pioneer species that allow for the subsequent sequential assembly of oral bacteria to form plaque biofilms, which can initiate diseases such as caries, gingivitis, and periodontitis [1,17]. Therefore, the ability of the SUD extract to inhibit *S. mutans* biofilm formation was determined.

This study found that SUD was able to inhibit *S. mutans* biofilm formation and disrupt *S. mutans* pre-formed biofilms. These data demonstrate a potential beneficial activity of SUD in the prevention of *S. mutans* biofilm mediated bacterial accumulation and warrants further investigation.

## Materials and Methods

### Sudantha herbal extract

Sudantha (SUD) extract is the active ingredient of a commercially available tooth paste Sudantha. It is a standardized crude proprietary mixture of 9 herbs formulated by a specialist panel of Ayurvedic clinicians and is quality controlled by high performance liquid chromoatography (HPLC). Its ingredients are: heartwood of cutch tree (*Acacia chundraWilld*.), malabar nut leaf (*AdhatodavasicaNees.),* Spanish cherry bark (*Mimusopselengi L*.), black pepper (*Piper nigrum L.*), pongam oil tree root (*Pongamiapinnata(L.) Pirerre*), Aleppo oak galls (*Quercusinfectoria Olivier*.), clove (*Syzygiumaromaticum L*.), myrobalan fruit (*Terminaliachebula Retz*.), and ginger (*Zingiberofficinale Roscoe).* This medicinal extract was stored at 4°C in the dark and freshly prepared to a stock concentration of 120 mg/ml a 12% ethanol diluted with 1% sucrose TYK medium (TYK+S). This stock concentration was then subsequently serially diluted with TYK +S medium to produce working experimental concentrations.

### Bacterial culture

*S. mutans* UA159 was obtained from the Darveau laboratory bacterial collection and grown planktonically overnight in TYHK (30 g/liter Trypticase soy broth, 5 g/liter yeast extract, 1 mg/liter menadione, 1 μg/ml hemin) or TYK broth at 37°C under anaerobic conditions (80% N_2_, 10% CO_2_, 10% H_2_). Overnight cultures were spun down and then resuspended with TYHK+1% sucrose (TYHK+S) or TYK+S to obtain a starter culture with an OD_600_ of 0.2–0.5.

### Biofilm inhibition

Starter cultures were grown with indicated concentrations of SUD in TYHK+S or TYK+S medium in 96-well flat bottomed plates in triplicate overnight (16–18 hours) at 37°C under anaerobic conditions; after which, crystal violet and susceptibility assays were performed. Wells with media alone served as positive controls and were used as the reference for total biofilm inhibition calculations, while wells without bacteria served as negative controls.

### Susceptibility assays

Total contents of the wells (planktonic and biofilm cells) were removed and resuspended in PBS. Then, ten-fold serial dilutions were plated onto blood agar plates and incubated at 37°C under anaerobic conditions for 24 hours before counting bacterial colonies. This experiment was performed 5 independent times in triplicate.

### Biofilm disruption assays

For biofilm disruption studies, starter cultures of *S. mutans* in TYK +S medium were grown in 96-well flat bottomed plates overnight at 37°C under anaerobic conditions. Afterwards the medium was discarded and replenished with indicated concentrations of SUD diluted in TYK+S medium in triplicates. Plates were then incubated for an additional 4 hours at 37°C under anaerobic conditions and crystal violet assays were performed. Biofilms grown in TYK+S alone served as positive controls and were used as the reference for total biofilm inhibition calculations, while wells without bacteria served as negative controls.

### Crystal violet assay

Biofilms were washed three times with deionized water to remove non-adherent cells. Then 0.1% crystal violet dye was added and incubated at room temperature for 10 minutes. Following incubation, the dye was removed and biofilms were washed three times with deionized water. Biofilms were then allowed to air dry completely and 30% acetic acid solution was added for 15 minutes. Solubilized dye was then mixed thoroughly and transferred into a new plate and read at an OD of 575 nm using a microplate reader. Percent biofilm inhibition was calculated by

$$Percent\,\;\,biofilm\;inhibition=([Control\;{OD}_{575}-Test\;{OD}_{575}]/Control\;{OD}_{575})\times 100\%$$

### Statistical analysis

Unpaired Student T-test was performed to determine significance for percent biofilm inhibition.

## Results

### SUD inhibits initial *Streptococcus mutans* biofilm formation

To assess the ability of SUD to inhibit *S. mutans* biofilm formation, planktonic *S. mutans* was grown overnight with half serial dilutions of SUD with the highest concentration at 3.75 mg/ml and lowest at 0.06 mg/ml. Overnight exposure to SUD showed dose dependent biofilm inhibition, where significant inhibition at 42% compared to control biofilms grown in media alone, occurred at 0.23 mg/ml SUD (p=0.002) and peak inhibition occurred around 92% at 0.94 mg/ml SUD (p<0.0001) (Figure 1A). Concentrations above 0.94 mg/ml SUD did not proceed to inhibit biofilm formation any further.

Enumeration of *S. mutans* cells, both biofilm and planktonic, exposed to 0.6–2.5 mg/ml SUD and grown overnight in a similar manner revealed a trend of dose dependent decrease in the total number of colony forming units (CFU) (Figure 1B). This suggests that inhibition of biofilms observed in (Figure 1A) may be partially due to reduction of cell viability.

### SUD disrupts pre-formed *Streptococcus mutans* biofilms

**Figure 2:**
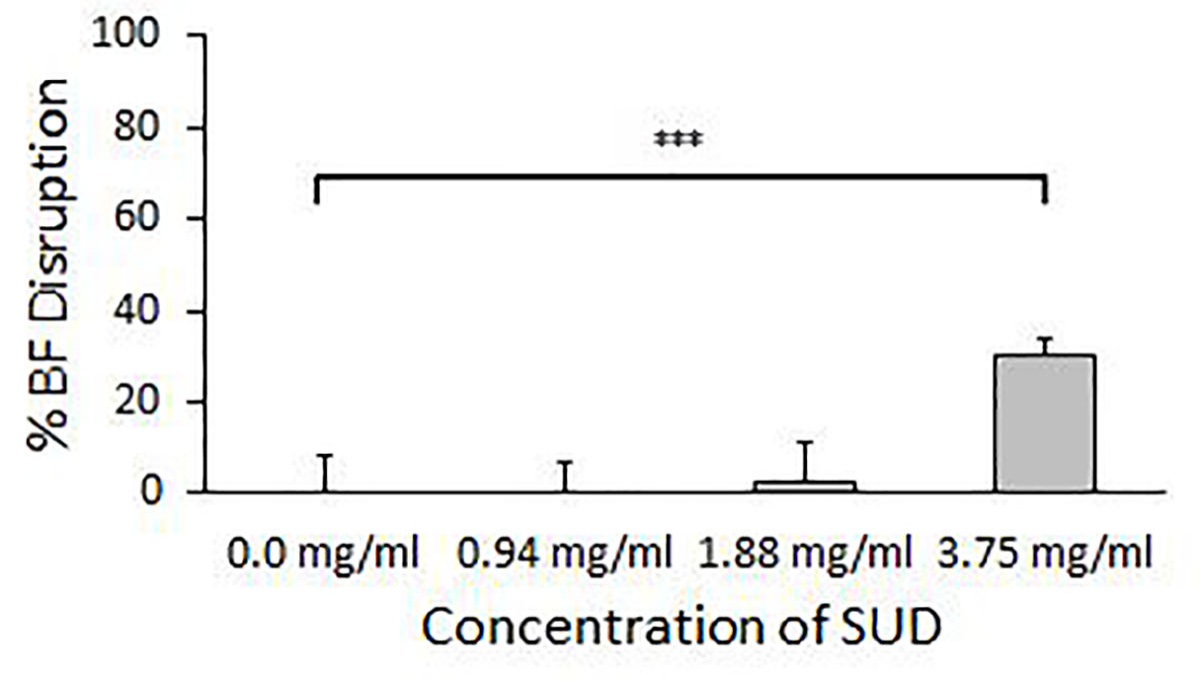
SUD disrupts pre-formed *Streptococcus mutans* biofilms. Percent biofilm disruption calculated from crystal violet assays of 4 hour exposure of SUD to overnight pre-formed *S. mutans* at indicated concentrations. Figure represents results from three independent experiments performed in triplicate. Controls were media alone and error bars represent standard deviations. Significance was determined by an unpaired Student T-test. (***P<0.001, **P ≤ 0.01, * P ≤ 0.05).

Subsequent to determining that SUD inhibited *S. mutans* biofilm formation, we assessed whether SUD also had the capability to disrupt pre-formed *S. mutans* biofilms*. S. mutans* biofilms grown overnight in 96-well plates were exposed to various concentrations of SUD for 4 hours. SUD disrupted pre-formed biofilms in a dose dependent manner, although biofilm release required higher concentrations of SUD compared to concentrations required for biofilm inhibition (Figure 2). Biofilm disruption was greatest with 3.75 mg/ml SUD which produced a 30% reduction in biofilm after 4 hours compared to controls with media alone (p<0.0001). The lower concentrations of 1.88 mg/ml and 0.94 mg/ml SUD examined did not show significant reduction in biofilms.

## Discussion

*Streptococcus mutans* is equipped with an array of virulence factors which help facilitate its adhesion, biofilm formation, and survival in a highly acidic environment [2,18]. As primary colonizers, streptococci play a large role in the formation of dental plaque, which is involved in the etiology of caries and periodontal diseases [1,17]. Of the several secreted products of *S. mutans*, glucosyltransferases (gtfs) and their glucan products are major virulence factors and play a significant role in biofilm production [18]. Glucosyltransferases B, C and D of *S. mutans* produce insoluble and soluble exopolysaccharides (glucans), a major component of the biofilm matrix, utilizing dietary sucrose as their primary substrate [18]. The collective action of these products enable bacteria in biofilms to survive harsh conditions such as mechanical shear forces, temperature extremes, physical properties of saliva, and exposure to antimicrobial agents in toothpastes and mouthwashes, as well as to antibiotics taken systemically.

Both *in vitro* and *in vivo* studies have reported successful application of herbal extracts and plant derived polyphenols on reduction of oral bacterial counts and inhibition of biofilm production [14,15,18–23]. Plant derived polyphenols are a class of natural compounds, including flavonoids, phenolic acids, and tannins, produced by plants in defense against invading pathogens and have been shown to inhibit *S. mutans* synthesis of glucan polysaccharides through the suppression of gtf genes [21]. Polyphenols have also been reported to be effective against biofilms produced by other oral pathogens, such as *Porphyromonas gingivalis* and *Enterococcus faecalis*, which are involved inflammatory diseases of the gums and root canal infections, respectively [22,23].

This pilot study found that SUD was able to inhibit initial *S. mutans* biofilm formation, as well as disrupt pre-formed biofilms. Biofilm inhibition was observed to be statistically significant with overnight exposure to 0.23 mg/ml to 3.75 mg/ml SUD. It is roughly estimated that an individual using a pea-sized amount of Sudantha® toothpaste will be exposed to 37.5 mg/ml SUD, which will be further diluted 10-fold with saliva in the mouth to ~3.75 mg/ml.Therefore, biofilm inhibition was observed at a fraction of the working pea-sized toothpaste concentration. Furthermore, reduction in total bacterial counts (planktonic and biofilm cells) after exposure to SUD overnight compared to controls suggest that initial biofilm inhibition may be partially due to antibacterial effects of the extract, which may occur as a direct or indirect effect of SUD. Alternatively, it is feasible that SUD may indirectly effect bacterial survival by suppression of gtf genes involved in exopolysaccharide production placing *S. mutans* into a more vulnerable state. This possibility will be examined in subsequent study.

Compared to biofilm inhibition, preformed biofilm disruption required higher concentrations of SUD at 3.75 mg/ml when exposed for 4 hours. The higher concentration required for disruption may be due to the decreased exposure time.

## Conclusion

Altogether, our data demonstrates that SUD is an effective agent against *S. mutans* biofilm *in vitro*. This data may partially explain one potential mechanism behind the efficacy of SUD gingival health that was observed in two randomized clinical trials by the significant reduction of gingival bleeding, dental plaque biofilm formation, and anaerobic bacterial counts. Furthermore, *in vitro* studies showed the ability of SUD to modulate host IL-8 inflammatory responses. Collectively, the data from this pilot study adds to the line of evidence behind the effectiveness of SUD in the maintenance of oral health and deserves further investigation.

## Limitations

The effect on mixed species biofilms was not examined and therefore these experiments address intra and not inter species adhesive interactions. Furthermore, due to the blended characteristic of the Sudantha® extract, it is difficult to assess which individual component within this mixture is mechanistically responsible for the anti-biofilm properties.

## Figures and Tables

**Figure1A:**
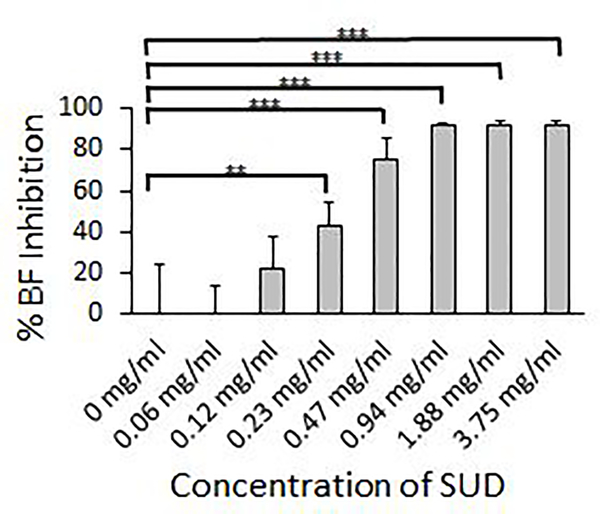
SUD inhibits initial *Streptococcus mutans* biofilm formation. Percent inhibition of biofilm formation calculated from crystal violet assays after 16–18 hour exposure to SUD at indicated concentrations. Figure represents results from three independent experiments performed in triplicate.

**Figure 1B:**
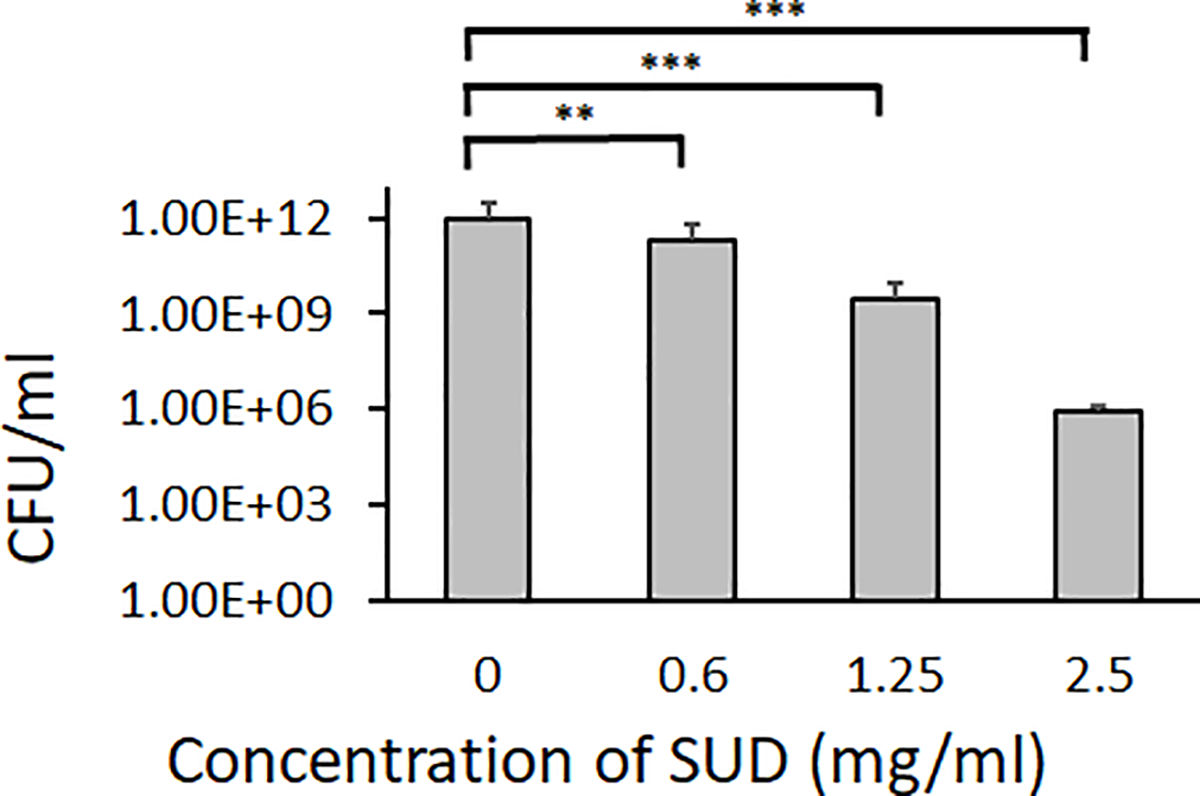
SUD inhibits initial *Streptococcus mutans* biofilm formation. Enumeration of total cells, both biofilm and planktonic, after 16–18 hour exposure to SUD performed five independent times in triplicate. In both experiments, controls were media alone and error bars represent standard deviations. Significance was determined by an unpaired Student T-test. (***P<0.001, **P ≤ 0.01, *P ≤ 0.05).

## Abbreviations:

CFU: Colony Forming Units
GTFs: Glucosyltransferases
HPLC: High Performance Liquid Chromatography
IL-8: Interleukin-8
SUD: Sudantha
TYHK+S: Trypticase Soy Broth, Yeast, Hemin, Vitamin K, 1% sucrose
TYK+S: Trypticase Soy Broth, Yeast, Vitamin K, 1% Sucrose
